# Maternal antenatal multiple micronutrient supplementation for long-term health benefits in children: a systematic review and meta-analysis

**DOI:** 10.1186/s12916-016-0633-3

**Published:** 2016-06-16

**Authors:** Delan Devakumar, Caroline H. D. Fall, Harshpal Singh Sachdev, Barrie M. Margetts, Clive Osmond, Jonathan C. K. Wells, Anthony Costello, David Osrin

**Affiliations:** Institute for Global Health, University College London, 30 Guilford St, London, UK; MRC Lifecourse Epidemiology Unit, University of Southampton, Southampton General Hospital, Tremona Road, Southampton, UK; Pediatrics and Clinical Epidemiology at Sitaram Bhartia Institute of Science and Research, B-16, Qutab Institutional Area, New Delhi, India; Faculty of Medicine, University of Southampton, Southampton General Hospital, Tremona Road, Southampton, UK; Childhood Nutrition Research Centre, Institute of Child Health, University College London, 30 Guilford St, London, UK

**Keywords:** Child, Micronutrients, Prenatal exposure delayed effects

## Abstract

**Background:**

Multiple micronutrient supplementation for pregnant women reduces low birth weight and has been recommended in low- and middle-income countries (LMICs) to improve child survival, growth and health. We aimed to review the evidence from long-term follow-up studies of multiple micronutrient supplementation beginning in the later first or second trimester.

**Methods:**

We searched systematically for follow-up reports from all trials in a 2015 Cochrane review of multiple micronutrient supplementation in pregnancy. The intervention comprised three or more micronutrients and the comparison group received iron (60 mg) and folic acid (400 μg), where possible. Median gestation of commencement varied from 9 to 23 weeks. Primary outcomes were offspring mortality, height, weight and head circumference, presented as unadjusted differences in means or proportions (intervention minus control). Secondary outcomes included other anthropometry, body composition, blood pressure, and cognitive and lung function.

**Results:**

We found 20 follow-up reports from nine trials (including 88,057 women recruited), six of which used the UNIMMAP supplement designed to provide recommended daily allowances. The age of follow-up ranged from 0 to 9 years. Data for mortality estimates were available from all trials. Meta-analysis showed no difference in mortality (risk difference –0.05 per 1000 livebirths; 95 % CI, –5.25 to 5.15). Six trials investigated anthropometry and found no difference at follow-up in weight-for-age *z* score (0.02; 95 % CI, –0.03 to 0.07), height-for-age *z* score (0.01; 95 % CI, –0.04 to 0.06), or head circumference (0.11 cm; 95 % CI, –0.03 to 0.26). No differences were seen in body composition, blood pressure, or respiratory outcomes. No consistent differences were seen in cognitive function scores.

**Conclusions:**

There is currently no evidence that, compared with iron and folic acid supplementation, routine maternal antenatal multiple micronutrient supplementation improves childhood survival, growth, body composition, blood pressure, respiratory or cognitive outcomes.

**Electronic supplementary material:**

The online version of this article (doi:10.1186/s12916-016-0633-3) contains supplementary material, which is available to authorized users.

## Background

Micronutrient deficiency is believed to affect approximately two billion people worldwide, with pregnant women being at particular risk because of their high metabolic demands [1]. The World Health Organization (WHO) considers micronutrient deficiency to be of particular concern in low-income countries, where women’s diets are likely to be deficient in both quantity and quality. The importance of micronutrients is becoming increasingly apparent, especially in resource-poor settings in which women may enter pregnancy with multiple micronutrient (MMN) deficiencies [2–4]. For example, 38 % of pregnant women have iron deficiency leading to anaemia [5] and 15 % have vitamin A deficiency causing night blindness and increasing the risk of infection [6, 7]. Impaired antenatal nutrition can affect fetal development and growth in the short term, subsequent growth and cognitive development in the medium term, and risk of chronic disease in the longer term [3]. There is good evidence that folic acid in pregnancy reduces neural tube defects and the mortality arising from them [8, 9]. While the evidence base is not substantial for iron and folic acid, a Cochrane review described a mean increase in birthweight of 57.7 g (95 % CI, 7.7–107.8 g) [10].

While there is evidence that MMN supplementation reduces the prevalence of small for gestational age (SGA) births [11], the primary rationale for recent recommendations to implement routine MMN supplementation for pregnant women in developing countries is that such birth weight increments will lead to reductions in childhood stunting and mortality [12, 13]. In addition to the immediate effects on health, growth in early childhood is important because of its association with adult health and human capital [14]. The hypothesis of the developmental origins of health and disease proposes critical or sensitive periods in early development in which environmental influences can have lasting effects on growth and physiology [15]. In humans, these critical periods are thought to occur predominantly in prenatal life and extend into early childhood. Suggested mechanisms include an interplay between environment (including maternal nutritional status), genes, and hormones, in which epigenetic regulation plays a part [3, 15, 16]. Evidence for the hypothesis comes mainly from observational studies showing associations between low birthweight and adverse adult health outcomes [17], from historical events such as the Dutch Hunger Winter [18], and from animal studies [19]. Long-term follow-up of children born during trials of maternal MMN supplementation offer a potentially stronger test of the hypothesis. If MMN supplements are to be recommended for pregnant women, it is important to know whether they lead to the sustained improvements in child health predicted by an increase in birthweight. In this review, we searched for reports that followed up the trials included in the 2015 Cochrane review [20], focusing on childhood mortality and health-related outcomes (growth, body composition, cardio-metabolic risk markers, cognition, lung function). We hypothesized that, compared with iron and folic acid, antenatal MMN supplementation would lead to longer-term improvements in health and survival.

## Methods

### Eligibility criteria

#### Types of report

We attempted to find all reports of follow-up of children born in the individual or cluster randomized controlled trials included in the 2015 Cochrane review. The review included 17 trials and excluded 97. The main reasons for exclusion were not meeting intervention and comparison group criteria, not assessing supplementation in a healthy population, and providing supplementation within food fortification initiatives [20].

#### Participants

Participants were children born in the trials in the 2015 Cochrane review [20] who had been subsequently followed up.

#### Intervention and comparison groups

The intervention group included women who had received multiple micronutrient supplements, defined as “containing three or more micronutrients” in pregnancy [20]. All intervention groups received iron and folic acid. For consistency, the comparison group chosen *a priori* was iron 60 mg and folic acid 400 μg, where possible, as this regimen is generally recommended for pregnant women [21]. We planned to consider all trials together, with the possibility of subgroup examination when supplementation regimens differed. Trials with co-interventions (for example, food supplements) were eligible provided that the co-intervention was similar in both allocation arms. If a second randomization was conducted after birth, we did not include arms that received MMN supplements in the comparison group.

#### Outcome measures

The primary outcomes were mortality (difference per 1000 livebirths), height and weight (as continuous WHO *z* scores, adjusted for age and sex), and head circumference (continuous difference), presented as unadjusted differences (intervention minus control). At a population level, risk differences may be more interpretable than odds ratios because they give a clearer impression of the effects of an intervention in terms of potential policy. When not reported, we calculated mean differences with 95 % confidence intervals. Mortality difference was either as described in the trial paper or follow-up report, or calculated from available trial profiles. Secondary outcomes included categorical proportions of stunting (< –2 *z* scores height-for-age), underweight (< –2 *z* scores weight-for-age), wasting (< –2 *z* scores weight-for-height) and low body mass index (BMI) (< –2 *z* scores BMI-for-age), descriptions of body composition (lean and fat mass, skinfold thickness), blood pressure, cognitive tests, and lung function. Cognitive and motor development were as assessed by trialists, including use of indexes such as the Bayley Mental Development Index, Bayley Psychomotor Development Index, Stanford-Binet Test, DENVER II Developmental Screening Test and Wechsler Intelligence Scale for Children. Effect modification by maternal nutritional status and child sex was reported when available. Birth outcomes were not considered as they had been reported previously [11, 22].

#### Search strategy and study selection

We searched PubMed, Web of Science and the Global Health Library for articles that included trial lead author names, location, trial number, or the given trial name (Additional file 1: Text S1). We contacted trial authors when eligibility was unclear and used a snowballing approach, searching citation lists and ‘related articles’. Study titles and abstracts were screened for eligibility by DD and JCKW independently. Differences were resolved by discussion. Full papers were obtained for potentially eligible citations. The results were assessed for multiple publications and one study was excluded. We did not assess reports of maternal outcomes. No exclusions were applied for age, dates of coverage, or language.

#### Data extraction and analysis

Publications from each trial were examined by two review authors (unconnected with the trial) independently (AC, BM, CHDF, DD, DO, HSS, and JCKW), using a pre-tested data extraction form. Differences were resolved by discussion. We contacted trial authors for clarifications and consolidated multiple reports from the same trial using data from the later follow-up. We used anthropometric *z* scores adjusted for age and sex (WHO reference ranges). Meta-analyses were conducted if sufficient reports were available, using the DerSimonian and Laird model. When numbers of deaths were provided for a sub-group of participants in a second age interval, we combined the data to produce an estimate of the mortality rate across both intervals using standard life-table techniques, and generated 95 % confidence intervals through a simulation approach. Outcome measures were risk differences per 1000 livebirths (mortality), differences in anthropometry *z* scores (height-for-age (HAZ) and weight-for-age (WAZ)) and absolute values (head circumference and blood pressure). We assessed heterogeneity using the I^2^ statistic. In view of anticipated contextual heterogeneity, we used a random effects model for all meta-analyses. Missing data were reported under attrition bias, but no further analyses were conducted. For cluster-randomised trials, we used reported cluster-adjusted estimates, irrespective of the method employed. If the analyses had not been adjusted for cluster design effect, we used a previously reported design effect from the trial paper to inflate the variance. Analysis was conducted in Stata, version 12 (StataCorp, College Station, TX, USA).

#### Risk of bias

Risk of bias for each study was assessed by two review authors (unconnected with the trial) independently (AC, BM, CHDF, DD, DO, HSS, and JCKW), using a pre-tested data extraction form. Differences were resolved by discussion and trial authors were contacted for clarifications. We used the same categorisation of bias of individual trials as in the Cochrane review 2015. We reported on bias in follow-up reports in the following categories: selection, attrition, reporting, performance and detection. We evaluated reporting bias by inspection of funnel plots for the primary outcomes of mortality, weight-for-age, height-for-age and head circumference.

#### Sub-group analysis

We performed sub-group analyses including trials that used UNIMMAP only and trials with different iron dosage (~30 or 60 mg) in control groups. Sensitivity analyses were not conducted.

## Results

### Description of trials with follow-up reports

Follow-up reports were identified for nine of the trials in the Cochrane review. In total, 88,057 women were recruited. The trials were spread geographically: two in Africa [23, 24], one in the Americas [25], and six in Asia [26–31]. All sites were rural, with the exception of Nepal Janakpur (urban and rural) and Guinea (semi-urban). Mean ages of mothers were similar and ranged from 21.5 (Nepal Janakpur) to 25.6 years (Indonesia). Mean maternal BMI, measured at recruitment during pregnancy, ranged from 19.3 kg/m^2^ (Nepal Sarlahi) to 24.1 kg/m^2^ (Mexico). Trial characteristics, with results summarized in the way in which they were presented in the trial papers, are shown in Table 1 and have been previously described in detail [20, 32].

**Table 1:** Description of trials with follow-up reports

Location	Trial	Randomisation scheme	Pregnant women allocated (MMN and Fe60 Fol groups only)	Mean duration from LMP to beginning of supplementation and when supplementation stopped^a^	Control content	Intervention content^b^	Mean maternal BMI (kg/m^2^)	Main results from original trial paper (intervention effect relative to comparison group)
Bangladesh JiVitA	West et al. [26]	Cluster	127,282 women under pregnancy surveillance; 44,567 pregnancies recruited in 596 clusters	Median at enrolment 9 weeks until 12 weeks after birth	Iron 27 mg, folic acid 600 μg	Vit A, thiamine, riboflavin, vit B_3_, vit B_6_, folic acid, B_12_, vit C, vit D, vit E, copper, iodine, iron, selenium, zinc	Mean BMI unknown; proportion < 18.5 40 %	Birthweight, + 54 g (95 % CI, 41–66 g) SGA RR, 0.98 (95 % CI, 0.96–1.01) Gestation, 0.30 weeks (95 % CI, 0.21–0.40 weeks) Neonatal mortality RR, 0.98 (95 % CI, 0.88–1.20)
Bangladesh MINIMat	Persson et al. [27]	Individual	1478	102 days until birth	Iron 60 mg, folic acid 400 μg and “usual” food supplementation	UNIMMAP and “usual” food supplementation	20.3	Birthweight in MMN, 2710 g (95 % CI, 2675–2745 g) Birthweight in control, 2665 g (95 % CI, 2631–2699 g) Gestation in MMN, 39.1 weeks (95 % CI, 38.9–39.3 weeks); gestation in control, 38.8 weeks (95 % CI, 38.6–39.0 weeks) Perinatal mortality RR, 0.95 (95 % CI, 0.59–1.5) Neonatal mortality RR, 1.1 (95 % CI, 0.64–2.0)
Burkina Faso	Roberfroid et al. [23]	Individual	1428	125 days until 3 months after birth	Iron 60 mg, folic acid 400 μg	UNIMMAP; also randomly assigned to receive either chloroquine 300 mg weekly or sulfadoxine 1500 mg and pyrimethamine 75 mg once in 2nd and 3rd trimesters as malaria chemoprophylaxis	20.9	Birthweight, + 41 g (95 % CI, –11 to 94 g), adjusted for malaria prevention and health centre SGA, 0.83 (95 % CI, 0.65–1.07), adjusted for malaria prevention and health centre Gestation, –0.04 weeks (95 % CI, –0.38 to 0.29 weeks), adjusted for malaria prevention and health centre Perinatal mortality OR, 1.78 (95 % CI, 0.95–3.32) Neonatal mortality OR, 2.1 (95 % CI, 0.78–5.64)
China	Zeng et al. [28]	Cluster	3811 women in 104 clusters	96 days until birth	Iron 60 mg, folic acid 400 μg	UNIMMAP	20.9	Birthweight in MMN, 3198 g; birthweight in control, 3174 g SGA in MMN, 16.9 %; SGA in control, 18.9 % Gestation in MMN, 39.8 weeks; gestation in control, 39.8 weeks PMR in MMN group, 50.9; PMR in control group, 36.9 NMR in MMN group, 12.4; NMR in control group, 10.7
Guinea Bissau	Kaestel et al. [24]	Individual	1403	158 days until birth	Iron 60 mg, folic acid 400 μg	UNIMMAP; if haemoglobin concentration < 70 g/L, given additional iron 60 mg daily; all women received insecticide-treated bednets, weekly antimalarial prophylaxis and malaria treatment if needed	23.3	Birthweight, + 53.0 g (95 % CI, –19 to 125 g) Perinatal mortality OR, 0.82 (95 % CI, 0.53–1.26) Neonatal mortality OR, 1.18 (95 % CI, 0.66–2.11)
Indonesia	Shankar et al. [29]	Cluster	31,290 women, 262 randomly assigned midwives	146 days until 90 days after birth	Iron 30 mg, folic acid 400 μg	UNIMMAP	Not recorded	Birthweight, + 21 g (95 % CI, –11 to 53 g) Perinatal mortality RR, 0.90 (95 % CI, 0.79–1.03) Neonatal mortality RR, 0.90 (95 % CI, 0.76–1.06)
Mexico	Ramakrishnan et al. [25]	Individual	873 children born in the trial were subsequently randomised to receive postnatal multiple micronutrients or iron and vitamin A supplements	69 days until birth	Iron 60 mg; no supplements on Sundays	Vit A, thiamine, riboflavin, niacin, vit B_6_, folic acid, vit B_12_, vit C, vit D3, vit E, iron, magnesium, zinc; no supplements taken on Sundays	24.1	Birthweight in MMN, 2981 g; birthweight in control, 2977 g SGA in MMN, 10.1 %; SGA in control, 11.8 % Gestation in MMN, 39.5 weeks; gestation in control, 39.6 weeks
Nepal Janakpur	Osrin et al. [30]	Individual	1200	112 days until birth	Iron 60 mg, folic acid 400 μg	UNIMMAP; if haemoglobin concentration < 70 g/L, given extra iron 60 mg daily and anthelminthic treatment; if symptoms of night-blindness, vitamin A 2000 μg	19.9	Birthweight, + 77 g (95 % CI, 24–130 g) Gestation, 0.2 weeks (95 % CI, –0.1 to 0.4 weeks) PMR in MMN, 49.0; PMR in control, 40.5 NMR in MMN, 30.6; NMR in control, 20.0
Nepal Sarlahi	Christian et al. [34] Christian et al. [31]	Cluster	14,036 women under pregnancy surveillance; 2007 pregnancies recruited in 169 clusters	80 days until 12 weeks after birth	Iron 60 mg, folic acid 400 μg, Vit A 1000 μg	Vit A, thiamine, riboflavin, vit B_3_, vit B_6_, folic acid, vit B_12_, vit C, vit D, vit E, vit K, copper, iron as ferrous fumerate, magnesium and zinc; albendazole was offered in 2nd and 3rd trimesters	19.3	Birthweight in MMN, 2659 g; birthweight in control, 2652 g SGA in MMN, 53.8 %; SGA in control, 51.7 % PMR in MMN group, 87.1; PMR in control group, 62.4 NMR in MMN group, 54.0; NMR in control group, 36.3

^a^Data from Margetts et al. [32]

^b^See Additional file 1: Table S1 for supplement constituents

*BMI* body mass index, *Fe60 Fol* iron 60 mg and folic acid supplement, *LMP* last menstrual period, *MMN* multiple micronutrient supplement, *NMR* neonatal mortality per 1000 livebirths, *PMR* perinatal mortality per 1000 livebirths, *RR* relative risk, *SGA* small for gestational age, *UNIMMAP* United Nations International Multiple Micronutrient Preparation

Six of the nine trials used the UNIMMAP supplement developed by UNICEF, the United Nations University, and WHO, and were designed to provide the recommended daily allowances. It contained vitamin A 800 μg, thiamine 1.4 mg, riboflavin 1.4 mg, niacin 18 mg, vitamin B6 1.9 mg, folic acid 400 μg, vitamin B12 2.6 μg, vitamin C 70 mg, vitamin D 5 μg, vitamin E 10 mg, copper 2 mg, iodine 150 μg, iron 30 mg, selenium 65 μg, and zinc 15 mg [33]. The Bangladesh JiVitA trial used the same micronutrients as UNIMMAP in similar doses. The supplement used in Nepal Sarlahi contained micronutrients in similar doses (with 60 mg iron), plus magnesium and vitamin K, but no selenium or iodine [34]. The supplement used in Mexico included iron 62.4 mg and magnesium 252 mg, and did not include copper, iodine or selenium [25]. In some cases, a comparison group of iron 60 mg and folic acid 400 μg was not available: Nepal Sarlahi included additional vitamin A [34], Mexico did not include folic acid [25], Indonesia used 30 mg iron [29], and Bangladesh JiVitA used 27 mg iron and 600 μg folate [26]. Supplement constituents are shown in Additional file 1: Table S1. Supplementation was initiated in early to mid-pregnancy, with a range of median commencement gestation across studies of 14 weeks (Table 1).

### Follow-up reports

We found 20 follow-up reports (Table 2 and Additional file 1: Figure S1). We divided the findings into five general categories: mortality [26, 27, 35–41], anthropometry [35, 38, 39, 41–44] and body composition [39, 44, 45], cardiovascular [39, 43, 46, 47], cognitive [37, 48–51], and respiratory [52]. Primary publications from the Bangladesh JiVitA and MINIMat trials included follow-up mortality data and were included in the list of follow-up reports. Meta-analyses were conducted for mortality, weight, height, head circumference and blood pressure outcomes.

**Table 2:** Description of follow-up reports

Location	Follow-up study	Age at follow- up	Eligibility/exclusion criteria for follow-up	Risk of bias in loss to follow-up	% follow-up in control group^a^	% follow-up in intervention group^a^	Outcome measures
Bangladesh JiVitA	West et al. [26]	6 months	None reported	5 infants lost to follow-up; assumed to be alive at 180 days	100 %	100 %	Mortality assessed at 1, 3 and 6 months
Bangladesh MINIMat	Persson et al. [27]	5 years	None reported	None	No reported losses	No reported losses	Mortality assessed at 5 years
	Khan et al. [42]	54 months (monthly to 1 year 3 monthly to 2 years)	Singletons	None; imputation used for missing data points	79.9 % of livebirths; 86.5 % of children with birth anthropometry	78.3 % of livebirths; 87.9 % of children with birth anthropometry	Length or height, weight
	Khan et al. [45]	54 months	Singletons with birth anthropometry	Children lost more likely to be first born and have lower birthweight	72.3 % children with birth anthropometry	70.9 % children with birth anthropometry	Mid-upper arm circumference, skinfold thickness, body composition by bioelectrical impedance
	Tofail et al. [48]	7 months	Subgroup of singletons born May 2002 to December 2003	Small but significant differences in children lost to follow-up; mothers had fewer children, higher haemoglobin and shorter gestation; no significant difference in these between allocation groups	Unable to calculate as the number of deaths is only provided for all allocation groups combined	Unable to calculate as the number of deaths is only provided for all allocation groups combined	Cognition (‘Support test’ and ‘Cover test’) Motor development (Psychomotor Developmental Index of Bayley Scales of Infant Development) Behaviour (modified Wolke in three domains: activity, emotion and vocalization)
	Hawkesworth et al. [46]	4 years (mean 4.6 years)	Singletons born at term with birth anthropometry	Children lost to follow-up more likely to have been firstborn and their mothers on average ~9 months younger with 6 months longer education	69.1 % of livebirths; 73.6 % of children with birth anthropometry	67.9 % of livebirths; 74.0 % of children with birth anthropometry	Blood pressure, kidney size by ultrasound (restricted to individuals born during the second half of MINIMat trial, June 2003 to June 2004), and glomerular filtration rate (restricted to individuals born during first y of MINIMat trial, June 2002 to June 2003)
Burkina Faso	Roberfroid et al. [35]	Monthly to 12 months and 30 months	Singletons	None	97.8 %	98.0 %	Length, weight, head, chest and mid-upper arm circumference
China	Wang et al. [41]	3 monthly to 12 months, 6 monthly to 30 months	Subgroup born in middle year of 3.5 years recruitment; congenital disease excluded (n = 3)	Not specified	84.9 % of selected subgroup	81.3 % of selected subgroup	Length, weight
	Li et al. [49]	3 monthly to 1 year	Subgroup born in middle year of 3.5 years recruitment	Small difference in Apgar scores	89.7 % of selected subgroup	88.6 % of selected subgroup	Cognition Mental and Psychomotor development (Bayley Scales of Infant Development)
	Li et al. [50]	7–9 years	Singletons; children who moved away were excluded	None	61.0 %^b^	60.7 %^b^	Cognition Mental development (Wechsler Intelligence Scale for Children)
Guinea Bissau	Andersen et al. [36]	0–2 years	None reported	Lost to follow-up different for maternal weight, age, height and parity; no difference between trial groups	77.1 %	77.8 %	Mortality by routine surveillance every 3 months up to 1 year and every 6 months after 1 year
Indonesia	Prado et al. [37]	42 months	2369 of 41,839 women enrolled randomly assigned to blood tests; children of 549 of these women who gave birth in a 6 month period	None reported	94.5 % of selected subgroup	92.8 % of selected subgroup	Cognition Adapted, validated tests for motor, language, visual attention/spatial, executive and socio-emotional function; adjusted analyses presented: confounders included ‘home observation and measurement of environment’ inventory score, child haemoglobin, mother’s mid-upper arm circumference, birth weight, MMN adherence
Mexico	Ramakrishnan et al. [38]	3 months 24 months	Singletons; children from antenatal trial subsequently randomised to receive MMN or iron + Vit A	Lost to follow-up younger mothers, more educated and less parous	24.9 % of livebirths in childhood randomisation control group; 69.3 % of children randomised in childhood control	26.2 % of livebirths in the childhood randomisation MMN group; 74.2 % of children randomised in childhood control	Height, weight, head circumference
Nepal Janakpur	Vaidya et al. [43]	2–3 years	None reported	Difference in lost to follow-up in maternal education, urban/rural residence and main household occupation	85.8 %	86.5 %	Height, weight, head, chest, waist, hip and mid-upper arm circumferences, skinfold thickness, blood pressure
	Devakumar et al. [39]	7–9 years	None reported	Small difference in lost to follow-up in maternal education and urban/rural residence	80.5 %	79.2 %	Height, weight, body composition by bioelectrical impedance, skinfold thickness, head, chest, waist, hip, mid-upper arm and upper leg circumferences, kidney dimensions, blood pressure
	Devakumar et al. [52]	7–9 years	None reported	Small difference in lost to follow-up in maternal education and urban/rural residence; small number (n = 5) with learning difficulties unable to complete spirometry	80.5 %	79.2 %	Spirometry, respiratory illness, asthma
Nepal Sarlahi	Stewart et al. [44]	6–8 years	3669 also in subsequent childhood trial of iron, folic acid and zinc	Lost to follow-up more likely to be of Pahadi (hill) ethnicity, have a literate mother and own a radio, and less likely to own cattle	89.2 %	91.2 %	Height, weight, mid-upper arm circumference, waist circumference, skinfold thickness
	Stewart et al. [47]	6–8 years	3673 also in subsequent childhood trial of iron, folic acid and zinc	Loss to follow-up did not differ between groups; large number did not complete all tests; children with missing data less likely to be of Pahadi ethnicity or to have had any schooling, and had slightly lower BMI	Overall 84.1 %; number of participants varied for each test or measurement	Overall 85.1 %; number of participants varied for each test or measurement	Metabolic syndrome BMI, waist circumference, blood pressure, HbA_1c_, urine microalbumin:creatinine, cholesterol, glucose, insulin, homeostasis model assessment
	Christian et al. [51]	7–9 years	In control group of subsequent iron and zinc pre-school trial; children followed up in this study represent 23 % of live births in relevant allocation groups in the original trial	Main loss to follow-up was exclusion of children taking part in postnatal supplementation trial; children lost more likely to be of Pahadi ethnicity, literate mother, own a radio and less likely to own cattle	75.2 % of subgroup	84.2 % of subgroup	Cognition Universal Non-verbal Intelligence Test Movement Assessment Battery for Children Finger-tapping test Executive Function (Stroop Numbers Test, backward digit span, go/no-go) Environment (Middle Childhood Home Observation for the Measurement of the Environment inventory) Maternal intelligence (Raven Coloured Progressive Matrices)
	Christian et al. [40]	6–8 years	None reported	None reported	96.1 %	96.0 %	Mortality

^a^Defined as total number measured as a proportion of the total number available for measurement (livebirths minus deaths), as a percentage

^b^The percentage excludes 1643 families who have moved out of the area

*MMN* multiple micronutrient supplement

### Mortality

**Fig. 1:**
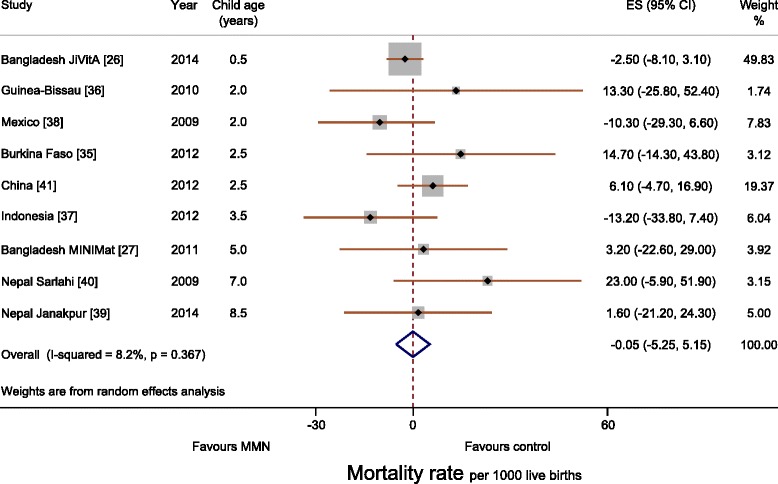
Forest plot showing mortality rate per 1000 livebirths (meta-analysis using a random effects model)

Follow-up reports from all trials systematically recorded and reported infant/child mortality as an outcome (Table 3). Meta-analysis showed no difference between intervention and control groups (risk difference, –0.05 per 1000 livebirths; 95 % CI, –5.25 to 5.15; I^2^, 8 %; Fig. 1). No difference by age was seen. Subgroup analysis including trials that used only the UNIMMAP supplement showed a risk difference of 3.41 per 1000 livebirths (95 % CI, –4.45 to 11.26; I^2^, 0 %; Additional file 1: Figure S2). Subgroup analysis for trials that used 60 mg iron in control groups yielded a risk difference of 4.51 per 1000 livebirths (95 % CI, –2.91 to 11.94; I^2^, 0 %), and trials that used approximately 30 mg iron in the control group yielded a risk difference of 0.41 per 1000 livebirths (95 % CI, –14.76 to 15.57; I^2^, 62 %; Additional file 1: Figure S3).

**Table 3:** Mortality outcomes

Location	Follow-up study	Deaths in control group^a^	Livebirths in control group	Deaths in intervention group^a^	Livebirths in MMN group	Mortality difference^a^ MMN – control, per 1000 livebirths (95 % CI)
Bangladesh, JiVitA	West et al. [26]	764 625 neonatal 139 at 29–180 days	14,142	741 626 neonatal 115 at 29–180 days	14,374	–2.5 (–8.1 to 3.1)^b^
Bangladesh, MINIMat	Persson et al. [27]	33 23 neonatal 10 post-neonatal to 5 years	612	34 25 neonatal 9 post-neonatal to 5 years	595	3.2 (–22.6 to 29.0)
Burkina Faso	Roberfroid et al. [35]	45 7 neonatal 38 post-neonatal to 30 months	644	55 11 neonatal 44 post-neonatal to 30 months	650	14.7 (–14.3 to 43.8)
China^c^	Wang et al. [41]	16 16 neonatal 0 in children born in 2004	1499	20 18 neonatal 2 in children born in 2004	1469	6.10 (–3.9 to 17.3)^b^
Guinea Bissau	Andersen et al. [36]	57 25 neonatal 9 at 29–91 days 10 at 92–365 days 13 at 366–730 days	519	65 29 neonatal 6 at 29–91 days 21 at 92–365 days 9 at 366–730 days	528	13.3 (–25.8 to 52.4)
Indonesia^c^	Prado et al. [37]	580 infant 17 in subsample	14,053	490 infant 12 in subsample	14,373	–13.2 (–33.4 to 7.1)^b^
Mexico^c^	Ramakrishnan et al. [38]	7 6 early deaths 1 in control group of childhood randomisation	460	4 4 early deaths	456	–10.30 (–29.30 to 6.60)
Nepal Janakpur	Devakumar et al. [39]	22 12 neonatal 6 post-neonatal 4 at 1–8 years	564	23 17 neonatal 4 post-neonatal 2 at 1–8 years	567	1.6 (–21.2 to 24.3)
Nepal Sarlahi	Christian et al. [40]	54 34 before 91 days 20 at 91 days to 7 years	773	81 52 before 91 days 29 at 91 days to 7 years	872	23.0 (–5.2 to 51.3)^b^

^a^Mortality figures given for the most recent follow-up. In the case of the Bangladesh MINIMat study, this was the trial paper

^b^The analysis accounts for clustering in relevant trials. Design effects of 1.15, 1.20 and 1.20 were predicted in the Bangladesh JiVitA, Indonesia and Nepal Sarlahi trials, respectively [26, 29, 34]; the China trial did not report a figure and we used a design effect of 1.20

^c^The follow-up report described a subgroup of children; confidence intervals were created using a simulation approach, as described in more detail in the methods section

### Anthropometry

Seven reports described anthropometry (Table 4). No differences were seen in any report at the most recent follow-up for WAZ, HAZ or head circumference, nor in any secondary anthropometric outcomes. Differences were seen at younger ages in two trials. In the Burkina Faso trial, greater mean WAZ (β, 0.13; 95 % CI, 0.04 to 0.23) and length-for-age *z* score (β, 0.13; 95 % CI, 0.02 to 0.24) were seen in the multiple micronutrient supplement group, and a lower proportion were stunted at 1 year (hazard ratio, 0.73; 95 % CI, 0.60 to 0.87), but there was no difference at 2.5 years of age [35]. In the Nepal Janakpur trial, greater mean WAZ (β, 0.14; 95 % CI, 0.00 to 0.27) was seen in the multiple micronutrient supplement group at a mean of 2.5 years, but there was no difference at 8.5 years (β, 0.05; 95 % CI, –0.09 to 0.19). Small increases were seen in head, chest, hip, and mid-upper arm circumferences at 2.5 years, but were also not present at 8.5 years [39, 43].

**Table 4:** Anthropometry, body composition and cardiovascular results

	Follow-up study	Weight difference (*z* scores)	% Underweight (< –2 *z* scores)	Height difference (*z* scores)	% Stunted (< –2 *z* scores)	Weight-for-height/length or BMI for age	% Wasted or low BMI (< –2 *z* scores)	Body composition and skin fold thickness	Body circumferences	Blood pressure	Other
Bangladesh MINIMat	Khan et al. [42] Anthro^a^	Calculated difference: 0 (–0.114 to 0.114)		Calculated difference: –0.01 (–0.13 to 0.11)	4.8 % (0.8–8.9) more stunting across all measurements	Calculated difference: Wt-for-ht *z* score, 0.01 (–0.09 to 0.11)					
	Khan et al. [45] Body comp^a^							Lean mass, 0.000 kg (–0.123 to 0.125 kg); fat mass, 0.001 kg (–0.058 to 0.061 kg); no difference in biceps, triceps, subscap or suprailiac skinfold thicknesses	Calculated difference: head, 0.02 cm (–0.20 to 0.24) MUAC, 0.11 cm (–0.04 to 0.26 cm)		
	Hawkesworth et al. [46]									Compared to 30 mg iron control adjusted for iron intervention dummy and food intervention variables: Systolic, 0.05 (–0.71 to 0.81); Diastolic, 0.55 (–0.10 to 1.20)	No difference in kidney volumes compared to 30 mg iron
Burkina Faso	Roberfroid et al. [35]	0.13 (–0.01 to 0.27)^b^	Hazard ratio in 1st year of life 0.84 (0.70, 1.02) ^c^	–0.02 (–0.18 to 0.14)^b^ Length for age was significantly higher in the first year, but this disappeared by 30 months	Hazard ratio in 1st year of life, 0.73 (0.60–0.87)^c^	0.20 (0.06–0.34)^b^ Wt-for-ht was lower initially but higher in MMN from ≥ 10 months	Hazard ratio in 1st year of life, 1.10 (0.90–1.35)^c^		MUAC for age z, 0.18 (–2.93 to 3.29)^b^		
China	Wang et al. [41]	Pooled (1–30 months) adjusted difference: 0.03 (–0.05 to 0.10)^d^	Pooled (1-30 months) adjusted. OR 0.95 (0.71, 1.29) ^d^	Pooled (1–30 months) adjusted difference, 0.02 (–0.07 to 0.10)^d^	Pooled (1–30 months) adjusted; OR, 0.82 (0.63–1.07)^d^	Pooled wt-for-length (1–30 months) adjusted difference, 0.03 (–0.05 to 0.11)^d^	Pooled wt-for-length (1–30 months) adjusted; OR, 0.89 (0.58–1.36)^d^				
Mexico	Ramakrishnan et al. [38]	Calculated difference: –0.1 (–0.34 to 0.14)		Calculated difference: 0.2 (–0.08 to 0.48)		Calculated difference: Wt-for-ht –0.2 (–0.42 to 0.02)			Calculated difference: head, 0.2 cm (–0.42 to 0.82)		
Nepal Janakpur	Vaidya et al. [43]	0.14 (0.00–0.27)	37.8 % in control, 36.6 % in MMN	0.08 (–0.06 to 0.22)	60 % in control, 56.7 % in MMN	Wt-for-ht 0.12 (–0.02 to 0.26)	5.5 % in control, 6.3 % in MMN	Triceps, 0.20 mm (0.00–0.40 mm)	Head, 0.24 cm (0.06–0.43 cm); MUAC, 0.24 cm (0.11–0.37 cm)	Systolic, –2.5 mmHg (0.47–4.55 mmHg); Diastolic, –1.5 mmHg (–3.1 to 0.4 mmHg)	
	Devakumar et al. [39]	0.05 (–0.09 to 0.19)	Not recorded by trial group	0.02 (–0.10 to 0.15)	Not recorded by trial group	BMI for age 0.04 (–0.09 to 0.18)	Not recorded by trial group	Lean mass, –0.05 kg (–0.43 to 0.34 kg); fat mass, –0.07 kg (–0.32 to 0.18 kg); no difference in biceps, triceps, subscapular and suprailiac skinfold thicknesses	Head, 0.18 cm (–0.02 to 0.38); MUAC, 0.04 (–0.15 to 0.23 cm)	Systolic, 0.02 mmHg (–1.02 to 1.05 mmHg); Diastolic, 0.13 mmHg (–0.93 to 1.19 mmHg)	No difference in kidney dimensions; exclusion of children with chronic or major illness made no difference
Nepal Sarlahi	Stewart et al. [44]	Calculated difference: –0.04 (95 % CI, –0.15 to 0.07)^e^	Calculated difference: 0.3 % (95 % CI, –5.4 to 6.0)^e^	Calculated difference: –0.02 (–0.13 to 0.09)^e^	Calculated difference: 3.7 % (–2.0–9.4)^e^	BMI for age calculated difference: –0.04 (–0.14 to 0.06)^e^		Triceps: FeFol + vit A, 5.84 mm; MMN, 5.9 mm; Subscap: MMN, 4.81 mm; FeFol + vit A 4.75 mm	MUAC: FeFol + vit A, 15.4 cm; MMN, 15.4 cm		
	Stewart et al. [47]								Waist: FeFol + vita A, 51.27 cm; MMN, 51.22 cm	Systolic: FeFol + vit A, 95.2 mmHg; MMN, 95.5 mmHg; calculated difference, 0.29 mmHg (95 % CI, –0.65 to 1.23)^d^ Diastolic: FeFol + vit A, 63.9 mmHg; MMN, 64.4 mmHg; calculated difference, 0.56 mmHg (95 % CI, –0.38 to 1.50)^d^	Non-fasting glucose: FeFol + vit A, 3.91 mmol/L; MMN, 3.86 mmol/L; LDL: FeFol + vit A, 1.89 mmol/L; MMN, 1.84 mmol/L; HDL: FeFol + vit A, 0.72 mmol/L; MMN, 0.70 mmol/L; at risk for metabolic syndrome: FeFol + vit A, 12.2 %; MMN, 11.9 %

Results are unadjusted differences unless otherwise stated. Calculated differences use results given in the paper to calculate the difference (intervention – control) in outcome and confidence intervals

*HDL* high density lipoprotein, *LDL* low density lipoprotein, *FeFol* iron and folic acid, *MMN* multiple micronutrient supplement

^a^Comparisons were made with the 60 mg iron, 400 μg folic acid group and “usual” food supplementation

^b^Parity, gestation, age at measurement, age at delivery, malaria treatment, health centre

^c^Malaria, health centre, parity, gestational age

^d^Mixed linear models. Fixed effects: treatment, age, gender, birth weight, preterm, parity, feed methods, time of stay at outdoor, illness or health in last month before the interview, mother’s height, educational level, occupation, number of supplement tablets consumed, and family socioeconomic status; random effects: village and individual subject

^e^We have adjusted for cluster randomized controlled trial, assuming a design effect of 1.20

Effect modification by maternal BMI or child sex was not found in any report with the exception of the Bangladesh MINIMat trial, in which stunting was greater in boys in the MMN group (Males, 7.8 %; 95 % CI, 2.0 to 13.6; Females, 1.8 %; 95 % CI, –3.8 to 7.3). A test for interaction was not reported.

**Fig. 2:**
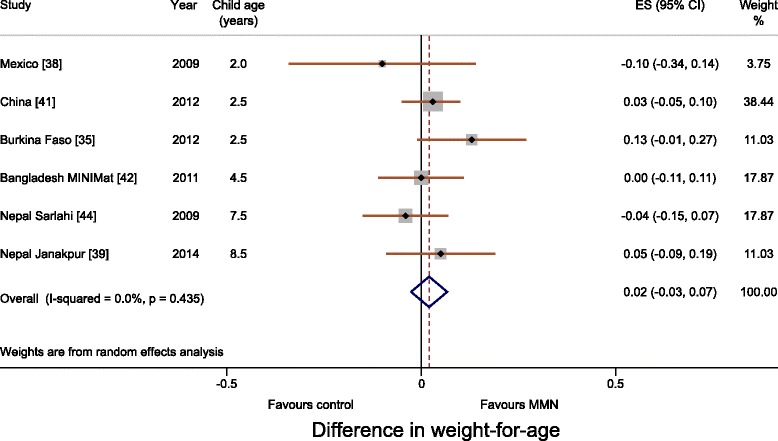
Forest plot showing weight-for-age (meta-analysis using a random effects model)

**Fig. 3:**
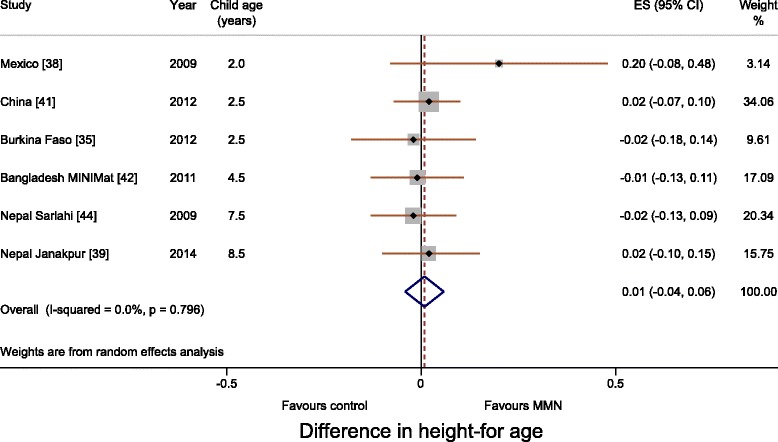
Forest plot showing height-for-age (meta-analysis using a random effects model)

**Fig. 4:**
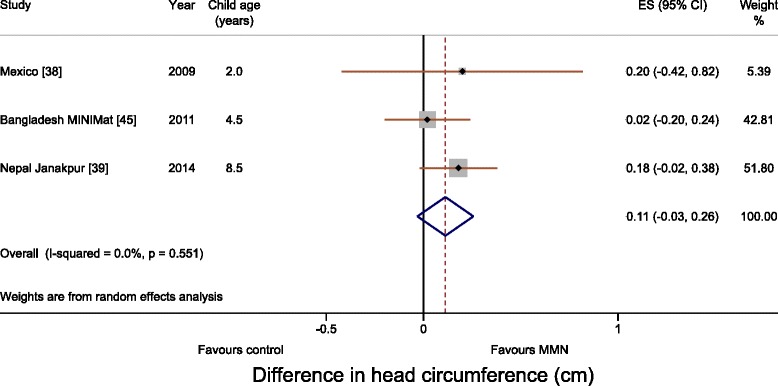
Forest plot showing head circumference (meta-analysis using a random effects model)

Meta-analyses for WAZ and HAZ showed no difference between multiple micronutrient and 60 mg iron and folic acid groups. The differences in WAZ and HAZ were 0.02 (95 % CI, –0.03 to 0.07; I^2^, 0 %; Fig. 2) and 0.01 (95 % CI, –0.04 to 0.06; I^2^, 0 %; Fig. 3), respectively. Meta-analysis for head circumference was possible for three trials (Mexico, Bangladesh MINIMat and Nepal Janakpur) and showed no difference (0.11 cm; 95 % CI, –0.03 to 0.26; I^2^, 0 %; Fig. 4). Subgroup analysis including UNIMMAP trials made little difference: WAZ 0.04 (95 % CI, –0.01 to 0.09; I^2^, 0 %), HAZ 0.01 (95 % CI, –0.05 to 0.06; I^2^, 0 %), and head circumference 0.11 cm (95 % CI, –0.05 to 0.26; I^2^, 10 %; Additional file 1: Figure S2).

### Body composition

The Bangladesh MINIMat trial found no difference in biceps, triceps, subscapular or suprailiac skinfold thicknesses. The Nepal Janakpur trial found an increase in triceps skinfold thickness at 2.5 years (0.20 mm; 95 % CI, 0.00 to 0.40 mm) [43], but no difference was found in any skinfold thickness at 8.5 years [39]. The Nepal Sarlahi trial found no difference in triceps or subscapular skinfold thickness at 7.5 years of age. Neither the Bangladesh MINIMat nor the Nepal Janakpur trial found a difference in lean mass or fat mass measured using bio-impedance [39, 45, 53, 54].

### Cardiovascular risk markers

Cardiovascular outcomes were only examined in trials from South Asia (Table 4). The Bangladesh MINIMat and Nepal Janakpur trials measured blood pressure, while the Nepal Sarlahi trial investigated metabolic syndrome (blood pressure, HbA_1c_, urine microalbumin:creatinine, cholesterol, glucose, insulin, homeostasis model assessment of insulin resistance). The Nepal Janakpur cohort showed a reduction in mean systolic blood pressure of 2.5 mmHg (95 % CI, 0.47 to 4.55) at 2.5 years of age, but no difference at 8.5 years (0.02 mmHg; –1.02 to 1.05) [39, 43]. The Bangladesh MINIMat cohort showed no difference at 4.5 years compared with a control group who received iron 30 mg and folic acid [46]. The Nepal Sarlahi trial found neither a difference in blood pressure at 7.5 years, nor a difference in other cardiovascular risk markers compared with a control group who received iron 60 mg and folic acid [47]. Meta-analysis of the three trials showed no difference in blood pressure: the difference in systolic blood pressure was 0.11 mmHg (95 % CI, –0.41 to 0.63; I^2^, 0 %), and in diastolic pressure 0.47 mmHg (95 % CI, –0.01 to 0.95; I^2^, 0 %; Additional file 1: Figure S4). Subgroup analysis for trials that used iron 60 mg in the control group was similar: systolic blood pressure difference 0.16 mmHg (95 % CI, –0.54 to 0.87; I^2^, 0 %), and diastolic 0.37 mmHg (95 % CI, –0.35 to 1.08; I^2^, 0 %; Additional file 1: Figure S3).

### Cognitive function

The Bangladesh MINIMat, China, and Indonesia trials all assessed subgroups of children: in Bangladesh, children born in a 19-month period, at 7 months of age [48]; in China, in the middle year of the trial up to 1 year of age [49] and at 8.8 years [50]; and in Indonesia, in women assigned to blood tests and whose children were born in a 6-month time period, at 3.5 years of age [37]. The Nepal Sarlahi study administered cognitive, motor and executive function tests at 7–9 years of age. Mean cognitive scores were a little lower for the MMN group compared to iron, folic acid and vitamin A (Universal Nonverbal Intelligence Test score, –2.4; 95 % CI, –4.6 to –0.2). Results of motor and executive function tests were mixed [51]. The Bangladesh MINIMat and China trials found no difference in motor or psychomotor scores [48, 49]. The Indonesia trial found an increase in motor ability in an adjusted analysis expressed as a fraction of the variation of the score (0.19; 95 % CI, 0.02 to 0.37) [37]. The Bangladesh MINIMat follow-up found no difference in problem solving or behaviour [48]. In the China trial, there were no differences at 3 or 6 months, but age-adjusted scores at 1 year were higher for mental development in the MMN supplementation group (1.20 points; 95 % CI, 0.32 to 2.08: equivalent to about 6 days of age) [49]. At 8.8 years of age, there were no differences in cognitive scores compared to folic acid or to iron and folic acid groups [50]. The Indonesia trial follow-up found no difference in visuospatial and visual attention, executive functioning, language ability, or socioemotional development [37].

For completeness, we mention stratified analyses. In the Bangladesh MINIMat trial, stratification by maternal BMI, combining the early and usual food groups together, showed an increase in Psychomotor Development Index in children whose mothers had been allocated to MMN supplements and had BMI < 18.5 kg/m^2^ (0.22 *z* scores; 0.01 to 0.42; interaction term, *P* = 0.05) [48]. In Indonesia, MMN supplementation was associated with greater motor ability (β = 0.35 *z* scores; 95 % CI, 0.01 to 0.69, interaction term, *P* = 0.04) and visual attention/spatial ability (β = 0.35; 95 % CI, 0.08 to 0.63, interaction term, *P* = 0.01) in children of undernourished mothers (MUAC, < 23.5 cm). These differences were equivalent to approximately 5 months of age [37].

### Respiratory

The Nepal Janakpur trial investigated lung function at 8.5 years of age. Spirometry data were obtained from 836 children, with 793 (95 %) achieving optimal results using American Thoracic Society/European Respiratory Society guidelines [55]. No difference in lung function was found between allocation groups: forced expiratory volume in the first second, –0.01 L (95 % CI, –0.04 to 0.02 L); forced vital capacity, –0.01 L (95 % CI, –0.04 to 0.02 L) [52].

### Assessment of bias

#### Trials

The trials were considered high quality and bias was not thought to be important. There was potential selection bias in the Guinea-Bissau trial as a result of inadequately concealed allocation [24], and potential attrition bias for the Nepal Janakpur and Mexico trials, in which exclusions prior to randomization were not reported [25, 30].

#### Follow-up reports

The trials were powered on the primary outcomes of gestational age and birthweight (and mortality, in the case of the Bangladesh JiVitA Indonesia trials) [29]. Follow-up reports described power or sample size calculations before data collection, with the exception of the Bangladesh MINIMat (cardiovascular) [46], Burkina Faso (anthropometry) [35] and Nepal Sarlahi (anthropometry and cardiovascular reports) follow-up publications [44, 47]. There was little statistical heterogeneity, with I^2^ values low for primary analyses (0–8 %). Some clinical heterogeneity was present as participants were from different countries and ages of follow-up varied. The intervention was the same in most cases, but (as described above) the Bangladesh JiVitA, Mexico and Nepal Sarlahi trials used slightly different multiple micronutrient formulations, and the Bangladesh JiVitA, Indonesia, Mexico and Nepal Sarlahi trials used different controls. Although choice of outcomes varied from one report to another, similar methods were used to assess similar outcomes.

#### Selection bias

Primarily a result of inadequate randomisation and allocation concealment, this has been covered in the 2015 Cochrane assessment [20]. An additional potential source of bias is selection of trials from the Cochrane review that did not show an increase in birthweight associated with MMN supplementation. The Cochrane review included 14 trials in its analysis of SGA. Of the five not considered here [56–60], one showed a significant reduction in SGA (Fawzi et al. [57], Tanzania; RR, 0.79; 95 % CI, 0.70–0.89). Supplement composition was substantially different in this trial, which compared a supplement containing eight vitamins and no minerals with a 60 mg iron and 250 μg folic acid control [57]. Meta-analysis of the trials included in our review showed an increase in birthweight of 30.2 g (95 % CI, 14.1 to 46.3), which is similar in magnitude to that found in previous meta-analyses [11, 61–63]. Similarly, three of the 11 trials included in the meta-analysis of neonatal mortality did not conduct follow-up studies. None of these trials showed a reduction in neonatal mortality. Meta-analysis of neonatal mortality rate for included trials produced an RR of 1.01 (95 % CI, 0.90 to 1.16).

#### Performance and detection bias

Participants and data collectors in all follow-up reports remained blind to allocation, with the exception of Guinea-Bissau, where this was not mentioned explicitly in the report.

#### Attrition bias

While all reports described loss to follow-up (Table 2), attrition bias was relatively small (0–29 %), except in the Mexico trial, in which just over half the children were seen at 24 months. The largest group lost were too old (>3 months) at the start of the follow-up. Excluding this group, follow-up rates were similar to those of the other reports. No important differences in loss to follow-up between allocation groups were reported, with the exception of China at 9 years, where study groups differed by school type, recent respiratory tract infection, mother’s occupation (farmer or other) and father’s level of education. These biases work in opposite directions and were accounted for in the analyses [50]. Where recorded, differences between children retained and lost to follow-up were small. Children lost to follow-up tended to have mothers with more education (Bangladesh MINIMat, Nepal Janakpur, Mexico and Nepal Sarlahi), lower parity and younger age (Bangladesh MINIMat and Mexico), and were more likely to live in an urban location (Nepal Janakpur), have differences in ethnicity and assets (Nepal Sarlahi), and lower birthweight and shorter gestation (Bangladesh MINIMat). Maternal age, weight, height and parity also differed in Guinea-Bissau, but the directions of these effects were not reported. Although most reports did not enumerate them, losses for individual outcomes also occurred: (>20 % loss to measurement) kidney volume and function in the Bangladesh MINIMat trial, body composition in the Nepal Janakpur trial, and fasting glucose, insulin and homeostasis model assessment in the Nepal Sarlahi trial.

#### Reporting bias

We could not make a definitive assessment of reporting bias as follow-up protocols were unpublished, but funnel plots for mortality, HAZ, WAZ and head circumference, using results from the most recent follow-up report, did not suggest publication bias for the primary outcomes (Additional file 1: Figure S5).

## Discussion

We found 20 follow-up reports for nine trials, covering a range of health outcomes. Nine studies reported on mortality, six on weight, six on height, and four on cognitive function, but there were few reports of other outcomes. Our hypothesis was not confirmed – we found no evidence that antenatal MMN supplementation, compared with iron and folic acid supplementation, led to improved survival, improved growth, lower blood pressure, or improved lung function in childhood. Potential improvements in cognitive outcomes were observed, but these were small and inconsistent and tended to be seen in subgroups.

### Quality of trials and limitations of the review

The trials had low risks of bias and were generally considered of high-quality [20]. The degree of loss to follow-up was not substantial, although in some cases only subgroups were followed up. Differential loss to follow-up between intervention and control groups did not appear to be an issue, and neither did selective publication, as most trials reported null findings. The evidence on antenatal MMN supplementation comes from a large sample and a substantial number of trials, many of which were coordinated. The trials considered here were spread geographically, although 13 of the 20 follow-up reports were from South Asia, which may have affected generalizability. Differences between the Nepal and Bangladesh reports emphasize the fact that variation can occur within similar populations.

The main limitation was that not all trials had published reports on follow-up. We cannot be certain whether a selection bias exists, but we found no indication of this. Follow-up reports have published null findings, rather than positive ones. If a publication bias does exist, it would not work in this direction. None of the trials had initially set out to observe childhood outcomes. While power calculations were performed prior to most follow-ups, trials were powered on birth outcomes and larger sample sizes may be required to detect small differences in childhood. Functional outcomes were measured at different ages and may not be comparable. We attempted to address this by using *z* scores, adjusted for age and sex, as primary outcomes. This was not always possible and limits inferences from the head circumference and blood pressure findings. We tried to make comparisons as similar as possible, but our findings could be vitiated by slightly different MMN and control supplement compositions in some trials.

### Mortality

Antenatal MMN supplementation was initially hypothesized to reduce mortality, but increases in neonatal mortality were suspected in some trials [64]. A meta-analysis did not find a difference in neonatal mortality overall, but raised concerns about increased early neonatal mortality (OR, 1.23; 95 % CI, 0.95 to 1.59). The 2015 Cochrane review found a reduction in stillbirths (0.91; 95 % CI, 0.85 to 0.98, *n* = 15), and no effect on perinatal (0.97; 95 % CI, 0.84 to 1.12, *n* = 12) or neonatal mortality (0.98; 95 % CI, 0.90 to 1.07, *n* = 11) [20]. The reduction in stillbirths is interesting and potentially important [65]. It depended on the analytical weighting (57 %) of the study by West et al. [26] and the use of a fixed effects model, and requires confirmation. A suggestion that MMN supplementation would prevent 43,715 annual child deaths (with 90 % coverage in 34 countries most in need) followed from the Lives Saved Tool model, based on evidence that reductions in the prevalence of SGA are associated with reduced stunting and subsequent child survival [12]. Our analyses did not identify evidence of an effect on child mortality and do not support the assumptions made in the Lives Saved Tool.

### Anthropometry and body composition

Overall, follow-up reports did not show differences in anthropometry or body composition. A transient improvement was seen in early life in the Burkina Faso and Nepal Janakpur trials, and there was a suggestion of increased stunting in Bangladesh, but these findings were not replicated in other reports. There was a consistent lack of effect on height. It is conceivable that MMN supplementation could have physiological effects that are not manifest in substantial anthropometric change. For example, transient greater weight in early childhood could have long-term benefits, even if not apparent in the short-term.

### Cognitive development

Small improvements in mental development were seen in China [49] at 1 year but not at 9 years, cognitive score was lower in Nepal Sarlahi [51], and the Bangladesh MINIMat and Indonesia trials found improvements in subgroups of mothers with poorer nutritional status only [37, 48]. Considering the number and range of tests conducted, antenatal MMN supplements did not appear to lead to a consistent cognitive benefit. When found, differences tended to be small and three of the five reports involved children under 4 years, in whom cognitive tests are less reliable than in older children. Further assessment of these cohorts is warranted.

### Other outcomes

Comparison of follow-ups did not confirm the impression of transiently lower systolic blood pressure at 2.5 years in the Nepal Janakpur trial, or of improved lung function in children seen in a similar trial of vitamin A supplementation from Nepal Sarlahi [66]. Only one trial considered other cardiovascular risk markers and found no effects.

### Inferences

On the basis of the reports we have reviewed, current evidence does not support changing the recommendation for routine antenatal supplementation from iron and folic acid to MMN formulations. It is possible – even probable – that the trial populations differed in their patterns of micronutrient deficiency. There was little evidence to suggest that this influenced the general findings. Although there is consistent evidence that antenatal MMN supplementation increases birth weight, none of the studies demonstrated convincingly that it benefitted offspring in terms of functional or health outcomes, and the directions and magnitudes of effect were similar for mortality and anthropometric outcomes across the study sites. The findings of the Cochrane review on which recent advocacy for routine antenatal MMN supplementation are based are supported by other meta-analyses that have shown an increase in mean birthweight of 22–54 g and corresponding reductions in low birthweight and SGA. As may be expected, the erosion over time of anthropometric differences observed at birth suggests that infants of women who received antenatal MMN supplements lost an advantage over the first few years. This could be the result of numerous environmental stresses over postnatal life. No other changes in anthropometry, gestation or mortality were found [11, 20, 22, 61–63]. However, improvement in later health outcomes does not necessarily depend on supplementation causing an increase in birthweight (or organ size). The mechanisms of action are likely to be multifactorial and follow-up reports suggest other mechanisms such as the effect of vitamin A on regulation of fetal lung growth, branching and alveolarisation [67, 68]. We also cannot rule out effects emerging later in life or in the next generation. From an evolutionary perspective, supplementing mothers could potentially benefit the woman herself, the index baby, or future offspring. Antenatal micronutrient supplementation has a role in women with a deficiency-related illness, and possibly in micronutrient deficiency itself, but population supplementation may need to start earlier, either in the first trimester or pre-conception, to include the period of rapid organogenesis and genome-wide epigenetic changes that follow fertilization, and be continued into childhood [16, 69, 70]. The formulations of MMN tested may also not be the optimum ones. We cannot rule out the possibility that combinations other than the ones tested might have positive outcomes. It is possible that additional micronutrients, or different doses, might result in functional benefits.

The annual cost of MMN supplementation in pregnancy (at 90 % coverage) in the 34 LMICs in which it would be most useful is estimated at (International)$ 472 million [12]. If, as our review suggests, initiating antenatal MMN supplementation in early/mid-pregnancy does not lead to the anticipated improvements in childhood function or health – the main current justification for recommending it – the opportunity costs to other programmes that do lead to improvements will need to be considered.

### Implications for future research

More evidence is needed, especially on cognitive development, cardiovascular risk markers and lung function, to adequately appraise the long-term effects of antenatal MMN supplementation. We recommend follow-up studies in more of the MMN trials. Further research into biological mechanisms by which an early advantage could be attenuated will help in our understanding of the intervention and in designing future trials.

## Conclusions

In summary, our review of published follow-up reports has not shown persisting effects of antenatal MMN supplementation during childhood, compared with iron and folic acid. Phenotypic and physiological differences may arise later in life or as more research is conducted, but the current evidence base is insufficient to support routine MMN supplementation in pregnancy at a population level in low- and middle-income countries.

### Abbreviations

BMI, body mass index; HAZ, height-for-age *z* score; MMN, multiple micronutrient; SGA, small for gestational age; WAZ, weight-for-age *z* score; WHO, World Health Organization.

## Additional file

Additional file 1: The search strategy, micronutrient constituents, PRISMA flow chart, sub-group analyses and funnel plots. (PDF 305 kb)
