# Supplementary material for: Maternal antenatal multiple micronutrient supplementation for long-term health benefits in children: a systematic review and meta-analysis
Source: BMC Med. 2016 Jun 16;14:90. doi: 10.1186/s12916-016-0633-3 (PMC4910255; doi:10.1186/s12916-016-0633-3)

Supplemental information

Maternal antenatal multiple micronutrient supplementation for long-term health benefits in children: a systematic review and meta-analysis

Devakumar D, Fall CHD, Sachdev HS, Margetts BM, Osmond C, Wells JCK, Costello A, Osrin D

Table of contents

Text S1: Search strategy ... 2
Table S1: Micronutrient constituents, dose per day ... 3
Figure S1: Flow chart showing number of articles found at each stage of search ... 4
Figure S2: Subgroup Meta-analysis UNIMMAP trials only ... 5
A) Mortality ... 5
B) WAZ ... 5
C) HAZ ... 6
D) Head circumference ... 6
Figure S3: Subgroup Meta-analysis Comparison to 60 mg and 30 mg iron groups. 7
A) Mortality reports with 60 mg iron comparison group ... 7
B) Mortality reports with 30 mg iron comparison group ... 7
C) Systolic blood pressure reports with 60 mg iron comparison group ... 8
D) Diastolic blood pressure reports with 60 mg iron comparison group ... 8
Figure S4: Forest plot showing blood pressure (negative values favour multiple micronutrients) ... 9
A) Systolic blood pressure ... 9
B) Diastolic blood pressure ... 9
Figure S5: Funnel plots for mortality, weight-for-age, height-for-age and head circumference ... 10

Text S1: Search strategy

We searched PubMed, Web of Science and Global Health Library with no date restrictions for:

1. (micronutrients or micronutrient*) AND trial author name (Bhutta ZA OR Brough L OR Christian P OR Dieckmann WJ OR Eneroth H OR Fawzi WW OR Friis H OR Gupta P OR Hawkesworth S OR Jarvenpaa J OR Kaestel P OR Khan A OR Osrin D OR Persson LA OR Ramakrishnan U OR Roberfroid D OR Rumiris D OR Shankar AH OR Sood SK OR Stewart C OR Sunawang UB OR Tatala SR OR Theobald GW OR Tofail F OR Vadillo-Ortega F OR Vaidya A OR West K OR Yan H OR Zagre NM OR Zeng L)

2. (micronutrient supplementation OR micronutrient*) AND location (sha?nxi china OR dhanusha nepal OR sarlahi Nepal OR sindh pakistan OR lombok indonesia OR dar es salaam tanzania OR east delhi india OR finland OR bissau, guinea-bissau OR harare zimbabwe OR hounde burkina faso OR cuernavaca mexico OR morelos mexico OR mexico city mexico OR university of indonesia OR indramayu java OR mpwapwa tanzania OR kongwa tanzania OR dodoma tanzania OR matlab Bangladesh OR niger)

3. Trial numbers (ISRCTN08850194, ISRCTN34151616, ISRCTN16581394, ISRCTN88625934, NCT00197548, NCT00642408, NCT00115271, NCT00860470)

4. Trial names (MINIMat, Nepal Nutrition Intervention Project, NNIPS, JiVitA)

In addition to the above searches, a snowballing method was used to retrieve lists of citing articles for all trials, using Web of Science and Scopus, and ‘related articles’ in PubMed, Web of Science and Scopus. We assumed that, as all the original trials were written in English, follow-up studies would be in English as well.

Table S1: Micronutrient constituents, dose per day

Micronutrients	UNIMMAP [a]	Bangladesh JiVitA [b]	Mexico [c]	Nepal Sarlahi [d]
Vitamin A (μg)	800	770 retinol activity equivalents	2150 IU	1000
Thiamine (mg)	1.4	1.4	0.93	1.6
Riboflavin (mg)	1.4	1.4	1.87	1.8
Folic acid (μg)	400	600	215	400
Niacin (mg)	18	18	15.5	20
Vitamin B_6 (mg)	1.9	1.9	1.94	2.2
Vitamin B_{12} (μg)	2.6	2.6	2.04	2.6
Vitamin C (mg)	70	85	66.5	100
Vitamin D (μg)	5	5	7.7	10
Vitamin E (mg)	10	15	5.7 IU	10
Vitamin K (μg)				65
Copper (mg)	2.0	1.0		2.0
Iron (mg)	30	27	62.4	60
Iodine (μg)	150	220		
Magnesium (mg)			252	10
Selenium (μg)	65	60		
Zinc (mg)	15	12	12.9	30

[a] UNICEF/WHO/UNU. Composition of a multi-micronutrient supplement to be used in pilot programmes among pregnant women in developing countries. New York: United Nations Children's Fund, 1999.
[b] West KP, Jr., Shamim AA, Mehra S, et al. Effect of maternal multiple micronutrient vs iron-folic acid supplementation on infant mortality and adverse birth outcomes in rural Bangladesh: the JiVitA-3 randomized trial. JAMA 2014;312(24):2649-58
[c] Ramakrishnan U, Gonzalez-Cossio T, Neufeld LM, et al. Multiple micronutrient supplementation during pregnancy does not lead to greater infant birth size than does iron-only supplementation: a randomized controlled trial in a semirural community in Mexico. Am J Clin Nutr 2003;77(3):720-5
[d] Christian P, Khatry SK, Katz J, et al. Effects of alternative maternal micronutrient supplements on low birth weight in rural Nepal: double blind randomised community trial. BMJ 2003;326(7389):571

Figure S1: Flow chart showing number of articles found at each stage of search

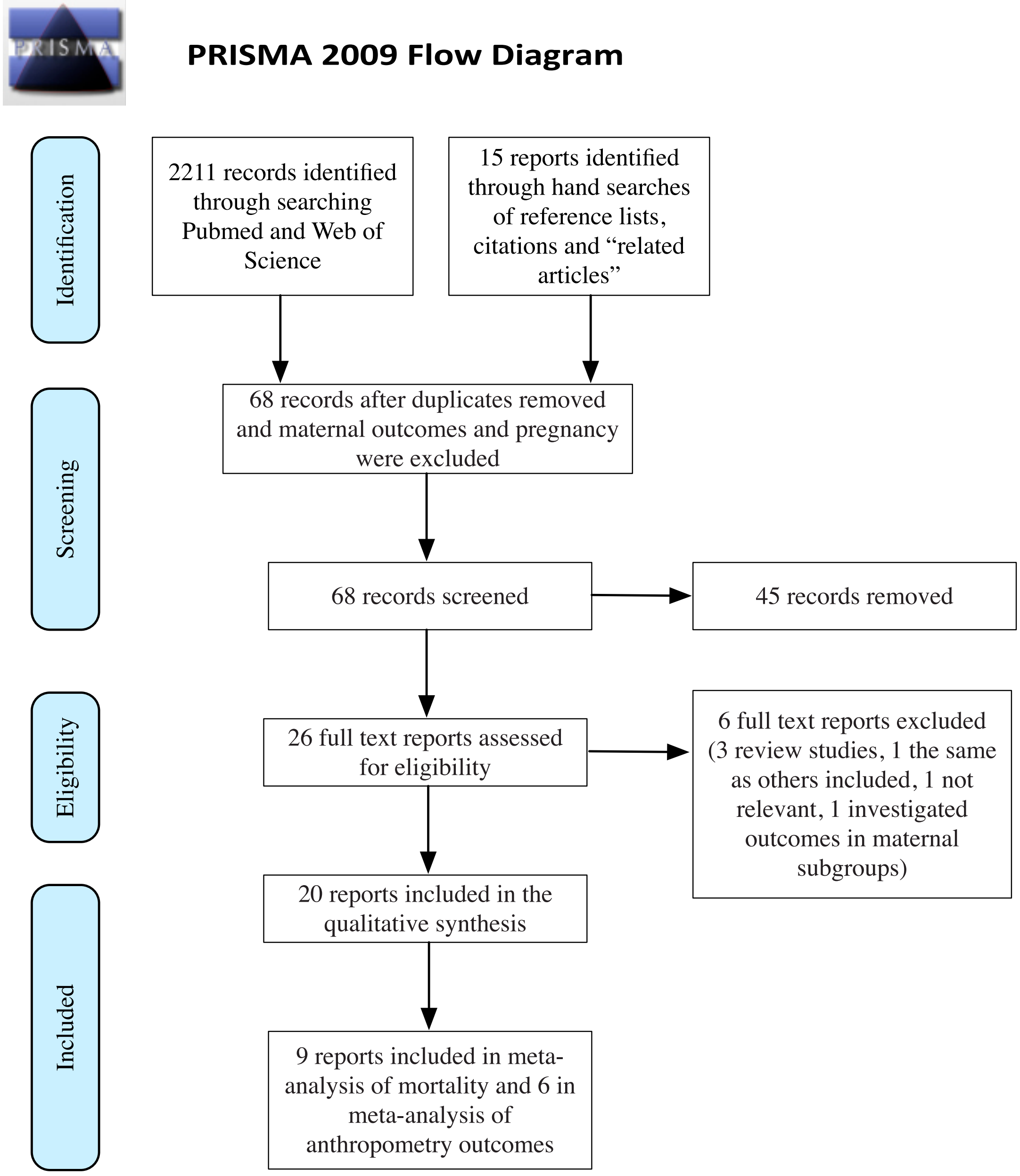

Figure S2: Subgroup Meta-analysis UNIMMAP trials only

A) Mortality

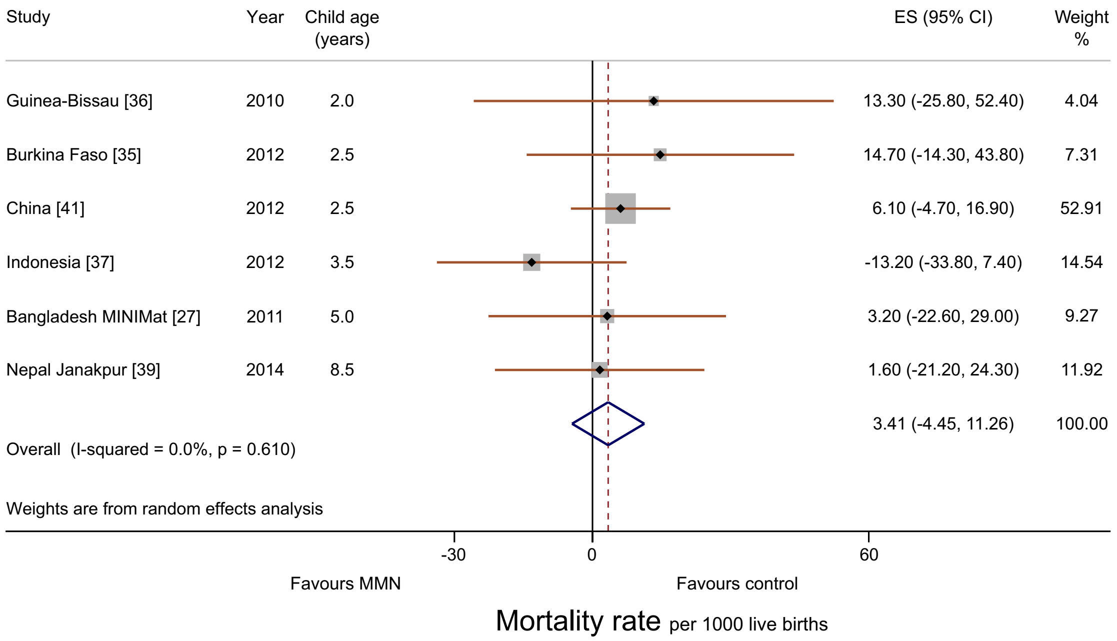

B) WAZ

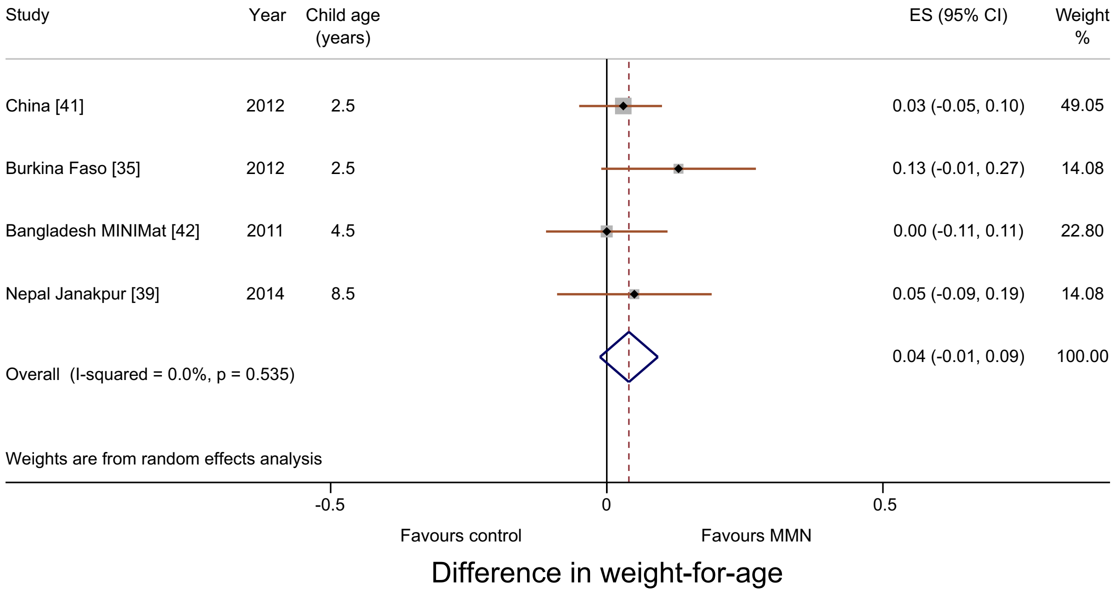

C) HAZ

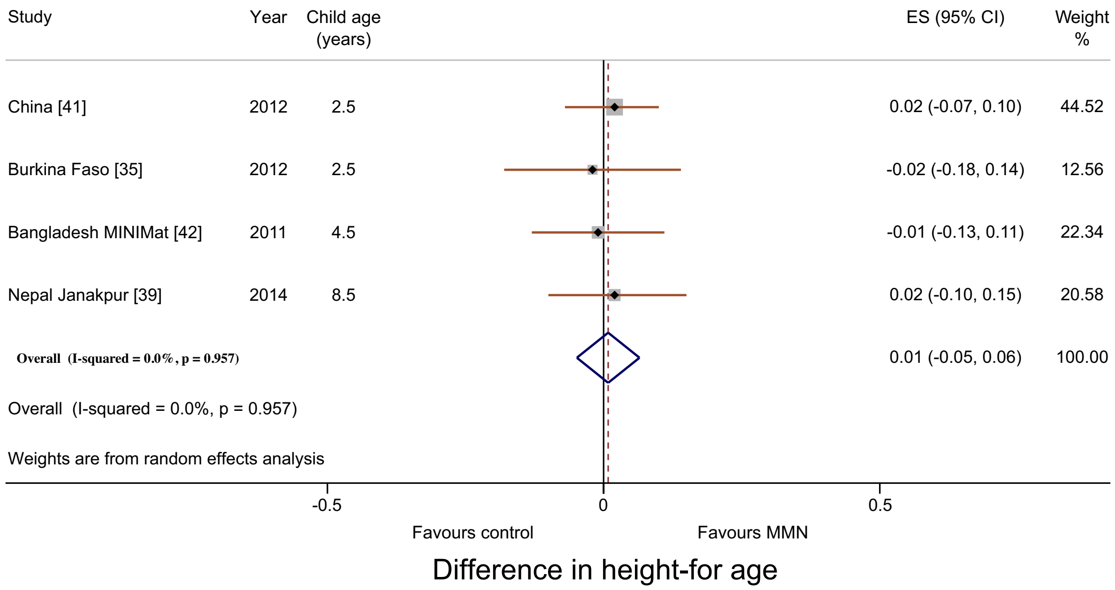

D) Head circumference

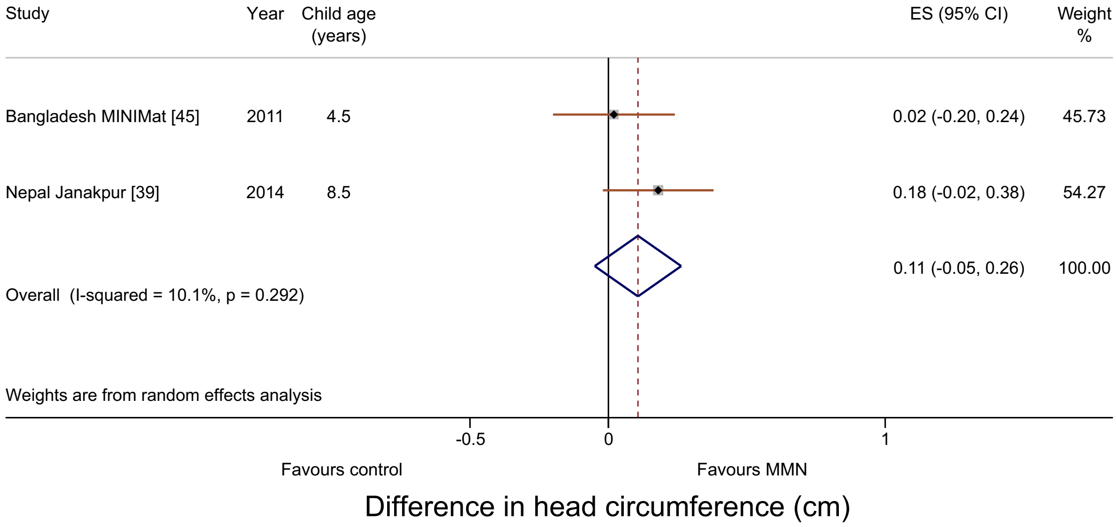

Figure S3: Subgroup Meta-analysis Comparison to 60 mg and 30 mg iron groups

A) Mortality reports with 60 mg iron comparison group

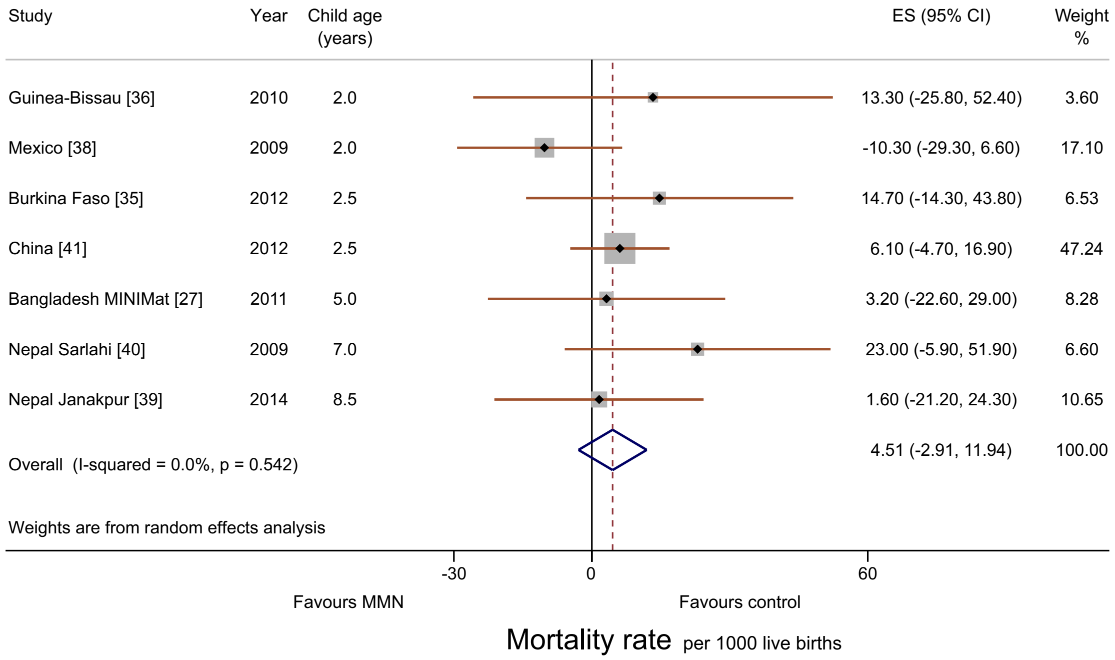

B) Mortality reports with 30 mg iron comparison group

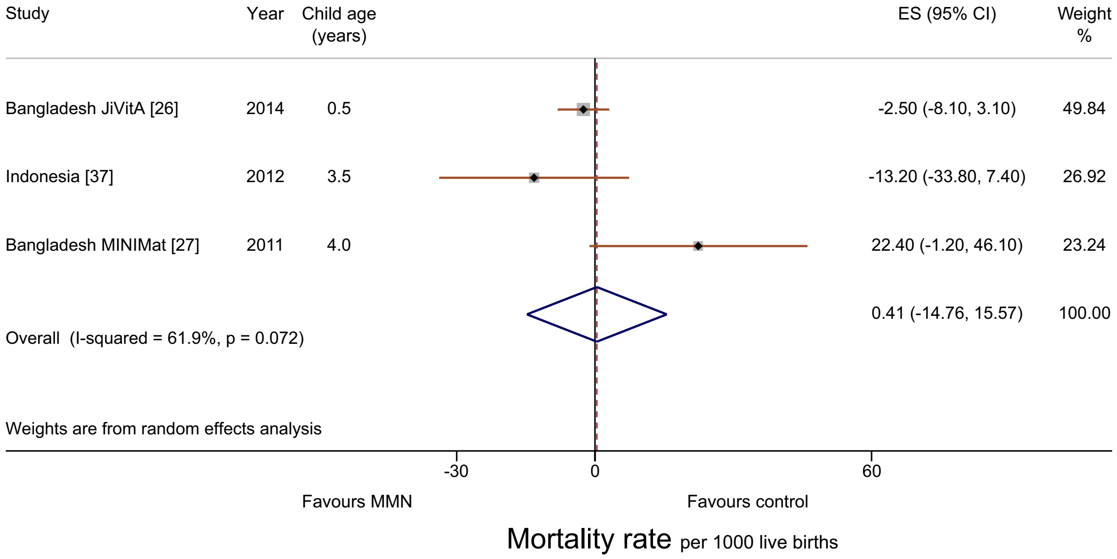

C) Systolic blood pressure reports with 60 mg iron comparison group

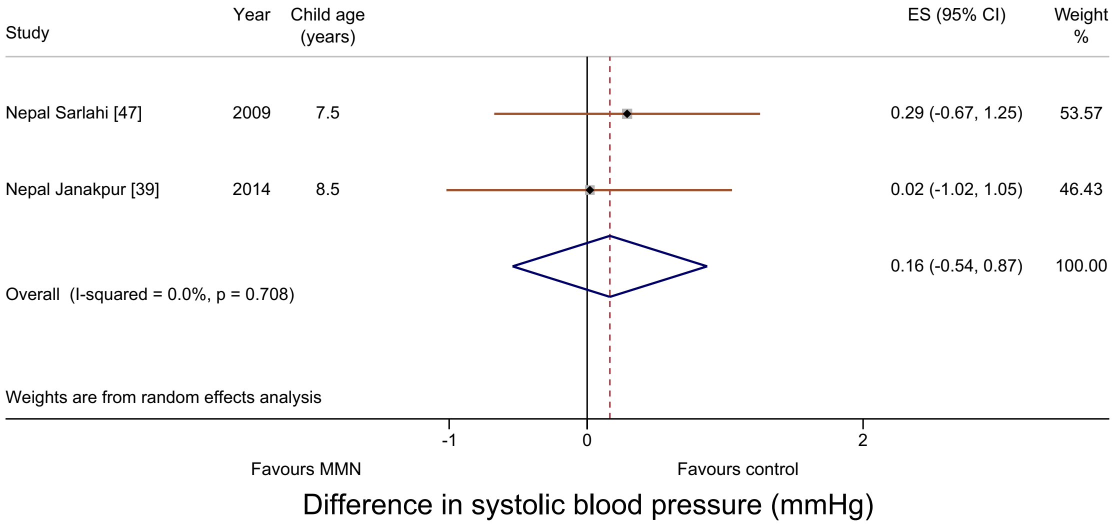

D) Diastolic blood pressure reports with 60 mg iron comparison group

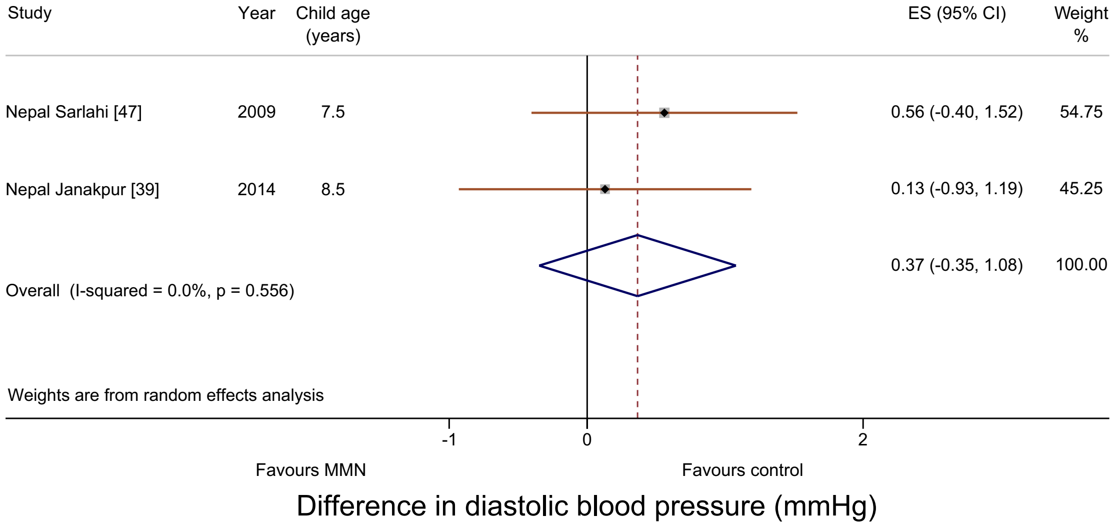

Figure S4: Forest plot showing blood pressure (negative values favour multiple micronutrients)

A) Systolic blood pressure

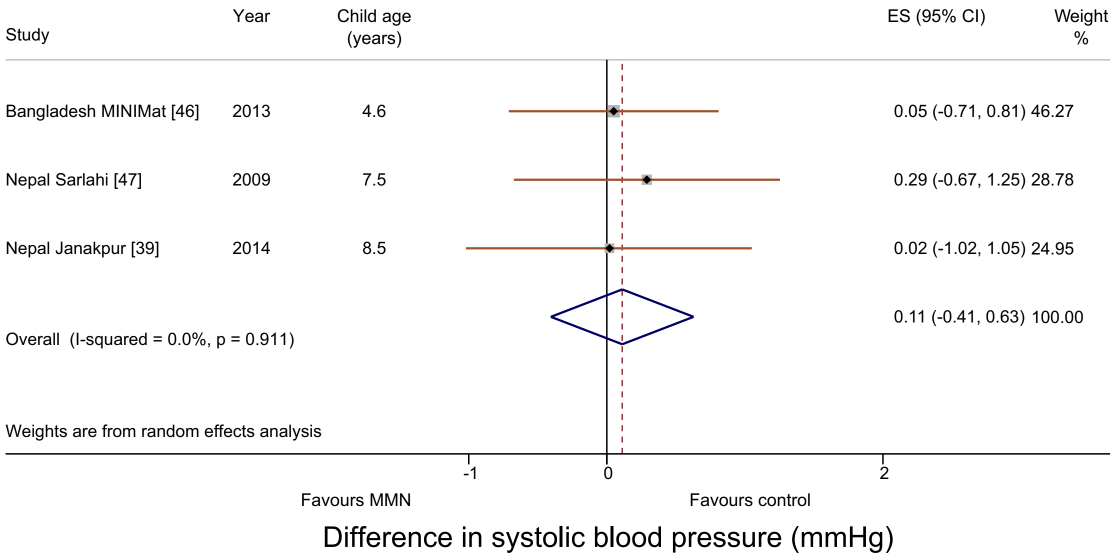

B) Diastolic blood pressure

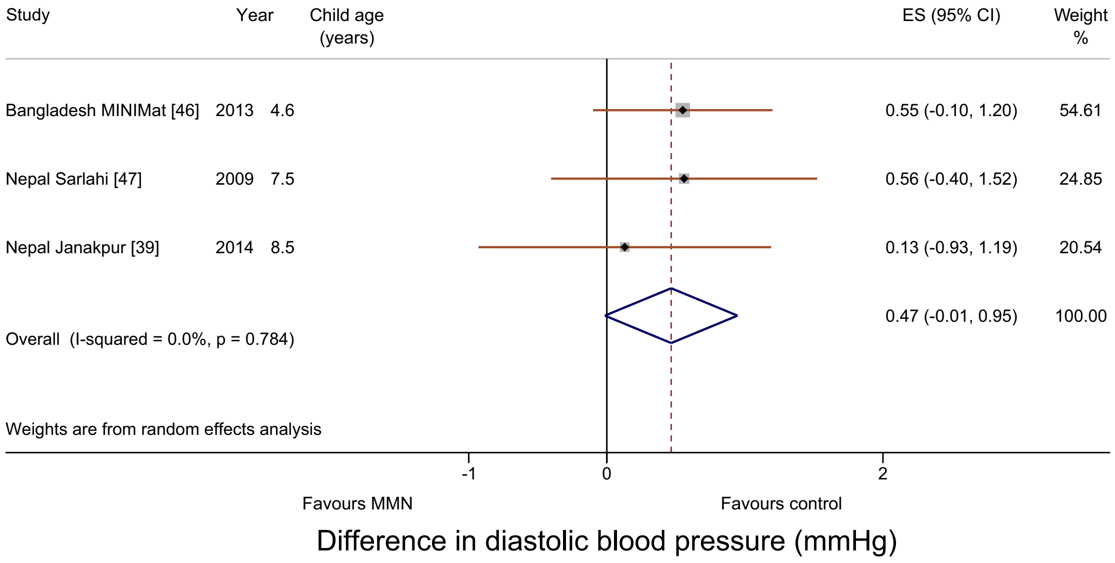

Figure S5: Funnel plots for mortality, weight-for-age, height-for-age and head circumference

a) Mortality

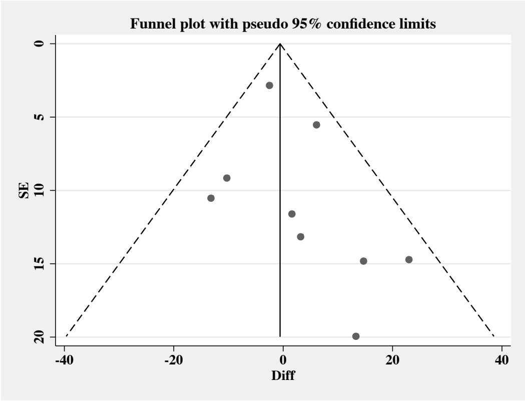

b) WAZ

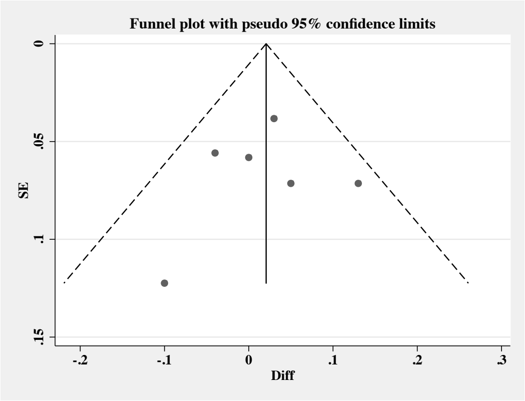

c) HAZ

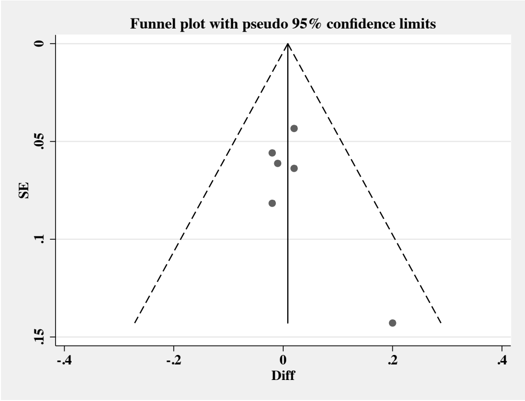

d) Head circumference

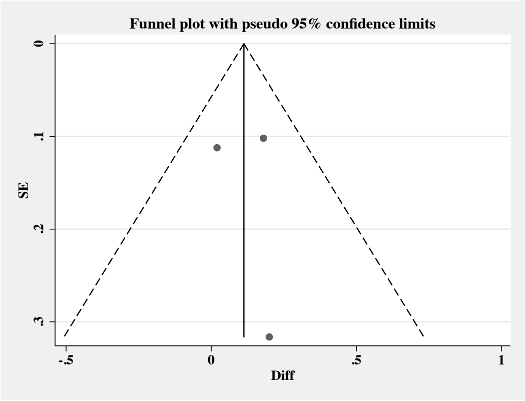

Supplement: Additional file 1: — The search strategy, micronutrient constituents, PRISMA flow chart, sub-group analyses and funnel plots. (PDF 305 kb) [file 12916_2016_633_MOESM1_ESM.pdf]
